# Impact of selenium-enriched *Ligilactobacillus salivarius* DACN818 on Zhacai: Insights from metabolomics and microbiomics

**DOI:** 10.1016/j.fochx.2026.103995

**Published:** 2026-05-19

**Authors:** Qingxu Guo, Jiajing Wang, Yue Li, Yi Wu, Dejun Liu, Xiaoyong Chen

**Affiliations:** a College of Food Science, Southwest University, Chongqing, 400715, China; b Chongqing Fuling Zhacai Group Co., Ltd., Chongqing, 408000, China; c Chongqing Agricultural Product Processing Technology Innovation Platform, Southwest University, Chongqing, 400715, China; d National Citrus Engineering Research Center, Citrus Research Institute, Southwest University, Chongqing, 400712, China

**Keywords:** Zhacai, *Ligilactobacillus salivarius*, Selenium enrichment, Volatile flavor compounds, Microbial diversity, Physicochemical characteristics

## Abstract

Selenium enrichment can modulate the metabolic activities of microorganisms, yet how such changes influence the quality characteristics of fermented foods remains poorly understood. This study investigated the effects of *Ligilactobacillus salivarius* DACN818 and its selenium-enriched variant (Se-DACN818) on the quality of Zhacai (Made from *Brassica juncea* var. *tumida* Tsen et Lee). Compared to the Control group at day 7, inoculation with Se-DACN818 increased the total content of volatile flavor compounds by 32.7%, with notable increases in alcohols, ketones, and esters. Additionally, compared to the DACN818 group, Se-DACN818 reduced pungent isothiocyanates by 55.37%; compared to the Control group, it decreased nitrite content by 53% and reduced brittleness by 17.6%. Metabolomics analysis revealed that Se-DACN818 enhanced the abundance of umami- and aroma-related compounds, including N2-maltulosylarginine and Ile-Asp-Glu. Correlation analysis showed significant positive associations between these umami- and aroma-related compounds and the abundance of *Ligilactobacillus*, whereas brittleness and protopectin content were significantly negatively correlated with the abundance of *Pediococcus*. This work provides a new strategy for enhancing the quality of fermented vegetables through selenium-enriched fermentation.

## Highlights

• *L. salivarius* DACN818 showed 98.3 ± 1.2% conversion of 2.5 μg/mL sodium selenite within 24 h.
• Se-DACN818 reduced the nitrite content in Zhacai by 53%.
• Se-DACN818 enhances the diversity and content of volatile flavors in Zhacai.
• Se-DACN818 reduced the content of pungent isothiocyanates in Zhacai.
• Aroma and umami compounds were positively correlated with *Ligilactobacillus* abundance.

## 1 Introduction

Selenium (Se), an essential trace element for living organisms, plays a critical role in regulating immune function, exerting antitumor activity, and supporting growth and metabolism. (Wan et al., 2018; Wang et al., 2024) Upon selenium enrichment, the metabolic activity of microorganisms can be altered, subsequently influencing the quality of fermented foods. Recent studies have shown that selenium-enriched *Saccharomyces cerevisiae* GDMCC 2.167 enhances the activity of key glycolytic enzymes (such as hexokinase, phosphofructokinase, citrate synthase), thereby accelerating the consumption of reducing sugars and promoting CO_2_ generation, which improves dough leavening and rheological properties. (He et al., 2023) In yogurt, selenium-enriched *Lactiplantibacillus plantarum* NML21 can promote the increase of 3-hydroxybenzoic acid and tetrahydrodipicolinic acid through ABC transporters and phenylalanine metabolism. (Wang et al., 2024) Similarly, selenium-enriched *Lactiplantibacillus plantarum* R enhanced the accumulation of glutamate and aspartate in Paocai, contributing to its umami flavor. (Zhou, Zheng, et al., 2021) Given their favorable safety and diverse probiotic functions, lactic acid bacteria (LAB) have emerged as a key focus in research on microbial selenium enrichment. (Yang & Yang, 2023).

Fuling Zhacai (Made from *Brassica juncea* var. tumida Tsen et Lee) is recognized as one of the three major pickled vegetables in the world, alongside French cornichons and German sauerkraut. However, the industry faces critical challenges due to its high salt content and limited health benefits, underscoring the urgent need for salt reduction and functional enhancement. Probiotic intervention has emerged as a promising strategy to address these issues. Previous studies have shown that that Zhacai harbors abundant LAB resources, including *Lactiplantibacillus plantarum*, *Lacticaseibacillus sakei*, and *Leuconostoc mesenteroides.* (Yang et al., 2021) These bacteria play essential roles in the quality formation of Zhacai, such as metabolizing carbohydrates to produce acids, inhibiting the growth of harmful microorganisms, reducing nitrite formation, slowing the loss of brittleness, and participating in amino acid metabolism. (Yu et al., 2023) Additionally, LAB are involved in the conversion of glucosinolates, thereby shaping the characteristic flavor profile of Zhacai. (Li, He, et al., 2023) These functions are closely associated with the metabolic activities of LAB. Nevertheless, the impact of selenium-enriched LAB on the quality of Zhacai has not been fully elucidated. *Ligilactobacillus salivarius* is a homofermentative LAB that produces lactic acid efficiently without generating acetic acid or carbon dioxide, and has simple cultivation requirements. It also has broad-spectrum antimicrobial activity, suggesting its potential for improving the quality and safety of fermented foods. (Li et al., 2021; Pidutti et al., 2018) However, research on the application of *Ligilactobacillus salivarius* in fermented vegetables, particularly in Zhacai, is still limited.

Therefore, this study aimed to investigate the effects of selenium-enriched *Ligilactobacillus salivarius* DACN818 (Se-DACN818) on the quality of Zhacai and the underlying mechanisms. The *Ligilactobacillus salivarius* DACN818 was selected for its high efficiency in converting sodium selenite. By comparing and analyzing changes in physicochemical properties, flavor compound profiles, and microbial community structure of Zhacai after fermentation, the regulatory effects of DACN818 and Se-DACN818 on its quality characteristics were elucidated. Furthermore, a correlation analysis between microbial communities and key quality indicators was constructed to reveal how Se-DACN818 modulates the quality of Zhacai, thereby providing a theoretical basis for the application of selenium-enriched strains in fermented foods.

## 2 Materials and methods

### 2.1 Bacterial strains and bacterial suspension preparation

DACN818 was isolated from traditional home-made Paocai in Jiangjin District (a selenium-enriched region in Chongqing, China). The strain is deposited in the China General Microbiological Culture Collection Center (CGMCC, Beijing, China) under the accession number CGMCC No. 29949.

A 200 μL frozen stock solution of DACN818 was inoculated into MRS broth (CM188, Beijing Land Bridge Technology Co., Ltd., Beijing, China) and incubated at 37 °C for 24 h. After three successive subcultures for activation, 5 mL the culture was centrifuged (4 °C, 4500 ×*g*, 10 min) to harvest the bacterial cells. The bacterial cells were washed three times with sterile saline, and then resuspended in sterile saline to adjust the optical density (OD_600_ = 1.0, corresponding to approximately 10^9^ CFU/mL). (Bhutto et al., 2015) Subsequently, a 2% (*v*/v) inoculum was transferred separately into MRS broth and MRS broth supplemented with 2.5 μg/mL sodium selenite (sterilized by filtration through a 0.22  μm membrane). Both cultures were incubated at 37 °C for 24 h. After centrifugation and washing, the final concentrations of DACN818 and Se-DACN818 suspensions were adjusted to OD_600_ = 1.0 for subsequent experiments.

### 2.2 Microscopic morphology observation of se-DACN818

DACN818 was inoculated into MRS broth containing 2.5 μg/mL sodium selenite and cultured at 37 °C for 24 h. After mixing, a 1.5 mL the cultured broth was centrifuged (4 °C, 4500 ×*g* for 10 min), and photographed. After digestion of the supernatant before and after conversion, the selenium content was determined using hydride generation atomic fluorescence spectrometry (HG-AFS) (AF-3320D, Beijing Beifen-Ruili Analytical Instrument (Group) Co., Ltd., Beijing, China). The conversion rate of sodium selenite by DACN818 was calculated according to formula 1. The bacterial cells were resuspended and fixed overnight in 2.5% glutaraldehyde solution, followed by centrifugation to harvest them again. Then they were sequentially washed with phosphate-buffered saline and dehydrated with a graded ethanol series and resuspended in isoamyl acetate for further dehydration. Finally, it was subjected to vacuum freeze-drying and sputter-coated with gold and observed under a scanning electron microscope (SEM, SEM5000X, Chinainstru & Quantumtech (Hefei) Co., Ltd., Anhui, China) to observe its morphological characteristics.

(1) $$\eta =\left(1-C_{t}/C_{0}\right)\times 100\%$$

*η*, Conversion rate of sodium selenite (%); *C*_*0*_, Initial selenium content in MRS broth (μg/mL); *C*_*t*_, Residual selenium content after conversion for 24 h (μg/mL).

### 2.3 Preparation of Zhacai with se-DACN818 fermentation

Zhacai (provided by Fuling Zhacai Group Co., Ltd., Chongqing, China) was mixed with sterilized and cooled fermentation nutrient solution (containing 0.75% white sugar, 0.75% rock sugar, and 4% sodium chloride) at a 1:1 (*w*/*v*) ratio in flexible packaging containers. The Control group was supplemented with 2% sterile saline, the DACN818 group received 2% DACN818 cell suspension, and the Se-DACN818 group was inoculated with 2% Se-DACN818 cell suspension. After mixing, residual air was removed from the packages, and the samples were fermented at room temperature in the dark for 7 days. After completion, samples were collected at designated time points for subsequent analysis.

### 2.4 Viable bacterial count

A 100 μL fermentation broth was subjected to ten-fold serial dilutions using sterile saline. Subsequently, a 100 μL appropriate dilution was spread onto MRS agar plates and incubated at 37 °C for 48 h, after which colonies were counted. (Tan et al., 2024).

### 2.5 Determination of physicochemical properties

The titratable acidity (TTA) was determined by acid-base titration and expressed as lactic acid equivalent. (Tan et al., 2024) The pH was measured using a pH meter (PHS-320, Chengdu Century Ark Technology Co., Ltd., Sichuan, China). Nitrite content was quantified using a nitrite rapid test kit (DS00503G, Beijing Kwinbon Biotechnology Co., Ltd., Beijing, China), and the absorbance of the final reaction solution was measured at 538 nm with a microplate reader (AMR-100, Hangzhou Allsheng Instruments Co., Ltd., Zhejiang, China). The content of protopectin (PP) was determined according to the carbazole colorimetric method and calculated as D-galacturonic acid equivalent. (Chen et al., 2024).

### 2.6 Determination of brittleness

The brittleness of Zhacai was determined using a texture analyzer (TA.XT Plus, Stable Micro Systems Ltd., Godalming, Surrey, United Kingdom). A cylindrical probe (P/5) was used in compression mode, with pre-test and post-test speeds set to 5.00 mm/s, a test speed of 4.00 mm/s, and a displacement distance of 5 mm. The maximum force (N) recorded from the puncture force curve was defined as the brittleness index. Following each measurement, the probe automatically returned to its initial position. Each group of samples was measured in five repetitions.

### 2.7 Microscopic morphology observation of Zhacai

The freeze-dried Zhacai samples were fractured and then mounted onto the sample platform using conductive adhesive, followed by sputter coating with a gold layer using an ion coater. The microscopic morphology of the fractured surfaces was then observed using a SEM (HITACHI SU8020, Hitachi High-Technologies Corporation, Tokyo, Japan).

### 2.8 Volatile flavor compounds analysis

Zhacai samples were weighed into a 30 mL SPME vial, followed by the addition of 5 mL saturated sodium chloride solution and 10 μL internal standard (0.1 μg/mL methyl heptanoate in anhydrous ethanol). The vials were sealed and extracted using a Supelco 50/30 μm DVB/CAR/PDMS fiber in a 60 °C water bath for 40 min, followed by desorption in the injection port at 250 °C for 5 min. Analysis was performed on an 8890-5977B gas chromatography–mass spectrometry system (Agilent Technologies, Inc., USA). GC conditions: a 30 m × 0.25 mm × 0.25 μm DB-5 ms low-bleed capillary column (Agilent Technologies, Inc., USA) was used; the initial oven temperature was maintained at 40 °C for 3 min, increased at 5.5 °C/min to 100.6 °C and held for 5 min, then raised at 10.7 °C/min to 250 °C and held for 2 min; splitless injection mode was applied with helium (99.999%) as carrier gas at a flow rate of 1.0 mL/min. The mass spectrometer was operated in electron ionization (EI) mode at 70 eV, with the ion source temperature maintained at 250 °C. Mass spectra were recorded over a scan range of *m*/*z* 35–500. Retention indices were calculated using a series of n-alkane standards (C7-C40, Beijing Solarbio Science & Technology Co., Ltd., Beijing, China). Compound identification was performed by comparing the acquired mass spectra with those in the NIST 23 library.

### 2.9 Microbial diversity analysis

A 2 mL fermentation broth was collected for microbial diversity analysis. Total genomic DNA was extracted using the E.Z.N.A.® Soil DNA Kit (Omega Bio-Tek, Inc., Norcross, USA). After verifying the quality and purity of the DNA, the V3-V4 hypervariable region of the bacterial 16S rRNA gene was amplified via PCR using barcode-indexed primers 338F (5′-ACTCCTACGGGAGGCAGCAG-3′) and 806R (5′-GGACTACHVGGGTWTCTAAT-3′). The resulting amplicons were purified and subsequently used to construct sequencing libraries, which were then sequenced on the Illumina Nextseq2000 platform.

### 2.10 Untargeted metabolomics analysis

A 1.0 mL of fermentation broth was mixed with an acetonitrile-methanol extraction solution (1:1) containing internal standards. After mixing, sonication, and incubation, the mixture was centrifuged. The resulting supernatant was evaporated to dryness and reconstituted in an acetonitrile-water solution. Following another round of sonication and centrifugation, the supernatant was collected for instrumental analysis. Analysis was performed using a UHPLC-Orbitrap system (Thermo Fisher Scientific, Waltham, United States) equipped with an HSS T3 column (Waters, Milford, United States). Separation was achieved using mobile phase A (water/acetonitrile, 95:5) and mobile phase B (acetonitrile/isopropanol/water, 47.5:47.5:5), both containing 0.1% formic acid, at a flow rate of 0.40 mL/min and a column temperature of 40 °C. Mass spectrometry analysis was conducted in positive and negative ion scanning modes (*m*/*z* 70–1050) with the following parameters: spray voltage 3500/−3000 V, sheath gas 50 arb, auxiliary gas 13 arb, ion transfer tube temperature 450 °C, and stepped collision energies of 20, 40, and 60 V. The raw data were processed using Progenesis QI software (Nonlinear Dynamics, Newcastle upon Tyne, UK) and matched against the HMDB (http://www.hmdb.ca), and Metlin (https://metlin.scripps.edu). Only secondary metabolites were included in the differential analysis.

### 2.11 Statistical analysis

Unless otherwise specified, all experiments were performed in triplicate, and the results are expressed as the mean ± standard deviation (SD). Data were analyzed using GraphPad Prism V9.5.0 (GraphPad Software, Inc., San Diego, USA). Raw data from microbial diversity and non-targeted metabolomics analyses were subjected to basic preprocessing, followed by data analysis and visualization via the Shanghai Majorbio Cloud Platform (https://cloud.majorbio.com). One-way analysis of variance (ANOVA) was employed to assess intergroup variability, and Tukey's HSD post hoc test was applied to determine significant differences in pairwise comparisons (*p* < 0.05).

## 3 Results and discussion

### 3.1 Conversion rate of sodium selenite and microscopic morphology of DACN818

**Fig. 1:**
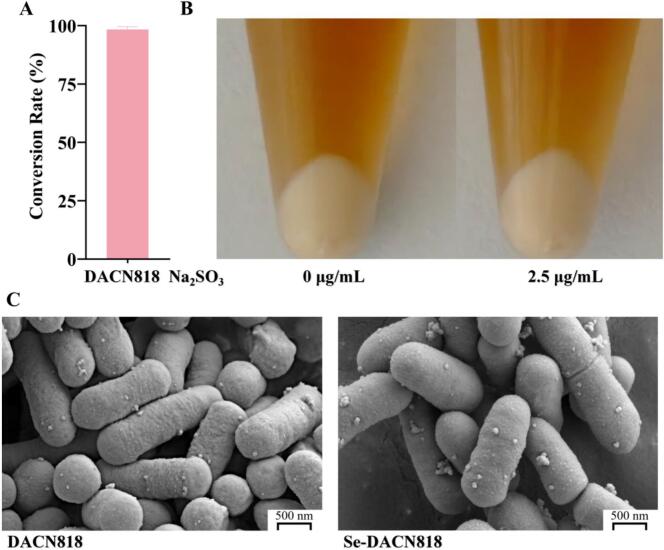
Sodium selenite conversion characteristics of DACN818. (A) Conversion rate of sodium selenite; (B) Photographs of cell pellets of DACN818 and Se-DACN818; (C) SEM micrographs of DACN818 and Se-DACN818.

As shown in Fig. 1A, DACN818 exhibited a high conversion efficiency of 98.3 ± 1.20% when cultured with 2.5 μg/mL sodium selenite for 24 h, confirming its high capability for sodium selenite conversion. At this concentration, no distinct red selenium nanoparticles (SeNPs) were observed in the precipitate of Se-DACN818 (Fig. 1B), suggesting that selenium was primarily transformed and accumulated intracellularly rather than forming visible extracellular SeNPs. This finding was consistent with previous reports indicating that LAB tend to incorporate selenium into intracellular metabolites at low selenite concentrations (<4 μg/mL), whereas extracellular red SeNPs are more likely to form at higher concentrations (>4 μg/mL). (Sun et al., 2022) SEM (Fig. 1C) revealed that both DACN818 and Se-DACN818 cells were typical rod-shaped morphology, with plump, dense structures and clear, intact contours. No shrinkage, collapse, or surface wrinkling was observed, indicating that selenium enrichment did not markedly alter the microstructure of DACN818.

### 3.2 Changes in viable bacterial counts

**Fig. 2:**
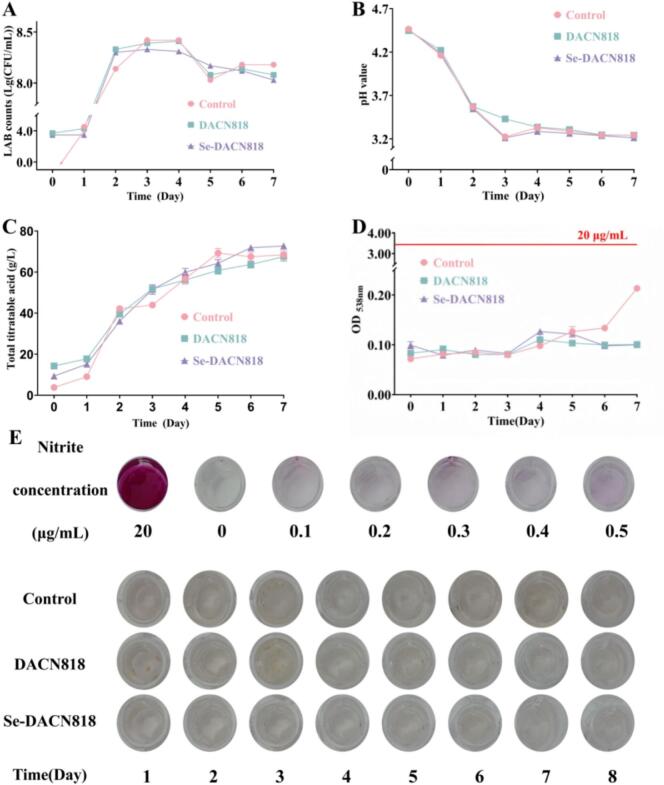
Changes in the key quality parameters. (A) Viable bacteria count; (B) pH; (C) TTA; (D, E) Nitrite content.

As shown in Fig. 2A, the trends in viable bacterial counts were similar across all groups. A rapid increase occurred within 1–2 days, reaching a peak on day 3, followed by a decline beginning on day 4 and subsequent stabilization. After stabilization, the viable counts in all groups remained above 8.03 lg CFU/mL. This pattern was consistent with previous findings in fermented vegetables. For example, a similar trend was reported in Zhacai fermented with *Lactiplantibacillus plantarum* LPP95, where viable counts peaked on day 3 before gradually declining. (Tan et al., 2024) In another study on Paocai fermented with a mixed starter culture containing *Levilactobacillus brevis*, *Weissella paramesenteroides*, and *Leuconostoc mesenteroides*, the bacterial counts peaked on day 4 in the spontaneous fermentation group and on day 7 in the inoculated group, followed by a decline or stabilization. (Luo et al., 2020) Notably, there was no significant difference in the changes of viable bacterial counts between the Se-DACN818 group and the DACN818 group, indicating that selenium enrichment did not alter the activity of DACN818.

### 3.3 Changes in pH and TTA

As shown in Fig. 2B and C, the pH values of all groups exhibited a gradual decreasing trend with prolonged fermentation time, while the TTA continuously increased. However, at the end of fermentation, no significant differences in the final pH or TTA were observed among the groups. Previous studies have reported that when Paocai is fermented using selenium-enriched *Lactiplantibacillus plantarum* R, the acid production capacity of the strain is significantly enhanced. (Peng et al., 2025) The proposed mechanism suggests that selenium enrichment may stimulate the activity of key metabolic enzymes in LAB, such as lactate dehydrogenase and acetate kinase (Chen et al., 2019; Liao & Wang, 2022). These findings suggested that the effect of selenium enrichment on the acid-producing properties of LAB is strain-specific.

### 3.4 Changes in nitrite content

Nitrite is a typical unsafe substance in fermented vegetables, the safety threshold for nitrite content in pickled vegetables is generally no more than 20 μg/g. (Yu et al., 2021) As shown in Fig. 2D and E, the nitrite content in Zhacai from all groups was below this limit during fermentation. The nitrite content in the Control group increased rapidly after day 4. In contrast, the nitrite content in both the DACN818 and Se-DACN818 groups began to decrease from day 4 onward. By day 7, the absorbance values corresponding to nitrite in the latter two groups decreased by 53% compared to the Control group. These results demonstrated that both DACN818 and Se-DACN818 possess a significant ability to reduce nitrite. Notably, previous studies have shown that selenium-enriched LAB may exhibit enhanced nitrite reduction capacity due to their stronger antioxidant activity. (Chen et al., 2019; Peng et al., 2025) However, in this study, DACN818 and Se-DACN818 showed no significant difference in nitrite degradation ability, which may be attributed to the fact that selenium enrichment did not alter the nitrite reduction metabolic pathway of the DACN818.

### 3.5 Changes in brittleness and PP content

**Fig. 3:**
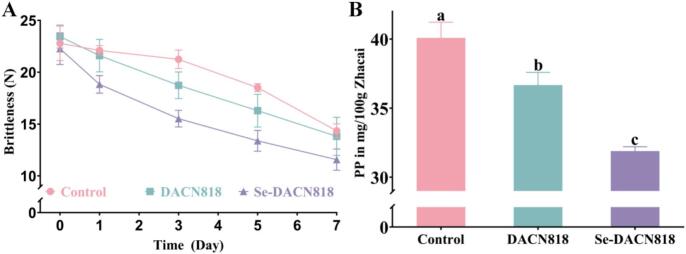
Changes in the textural characteristics. (A) Changes in brittleness; (B) PP content.

PP, a major structural component of plant cell walls, is closely associated with the brittleness of vegetables. It is typically degraded into water-soluble pectin by pectinase and acid, leading to loosening and softening of the cell wall structure and a decrease in brittleness. (Anderson & Pelloux, 2025) As shown in Fig. 3A, the brittleness of Zhacai decreased over the fermentation period in all groups, with obvious differences among the groups. The Control group exhibited the slowest decrease in brittleness, followed by the DACN818 group, while the Se-DACN818 group showed the most rapid decrease and the lowest final brittleness, with a 17.6% reduction compared to the Control group. This result was consistent with the analysis of PP content at day 7 (Fig. 3B), indicating that the greater reduction in brittleness in the Se-DACN818 group can be attributable to a greater amount of PP degradation.

### 3.6 Microscopic morphology of Zhacai

**Fig. 4:**
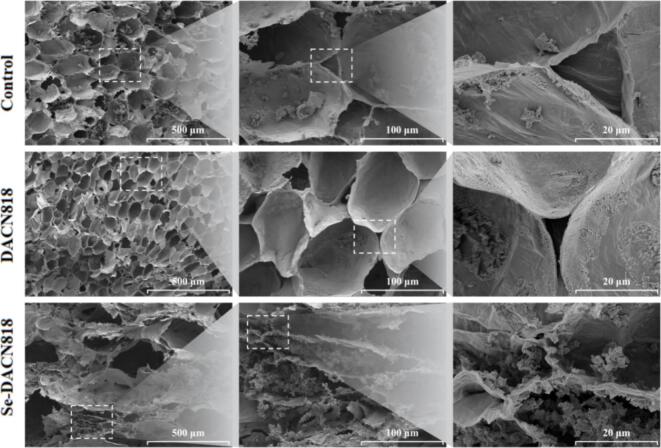
Microscopic morphology of Zhacai

The SEM enables the observation of cell wall integrity, pore structure morphology, and differences in the micromechanical support structures of tissues. Generally, more intact cell walls and tighter structures provide greater mechanical support, contributing to superior brittleness. (Bhuiyan et al., 2026) As shown in Fig. 4, although the Control group exhibited relatively large intercellular pores, its cell walls were thicker and the fracture surfaces were relatively intact, with few structural fragments on the surface, indicating a more preserved cellular architecture for load-bearing. The DACN818 group showed regular cell pores but also exhibited localized cell wall thinning and slight collapse, with the fracture surfaces appearing torn, suggesting a potential reduction in the compressive strength of the cell walls. The Se-DACN818 group displayed the loosest structure, characterized by severe cell collapse, an uneven surface, and a significant loss of structural integrity. These morphological changes are likely closely associated with the degradation of PP. (Satapathy et al., 2020).

### 3.7 Volatile flavor compounds analysis

Volatile organic compounds are key elements in the flavor formation of fermented vegetables. (Wang et al., 2022) As shown in Table 1, the Se-DACN818 group exhibited the most complex profile of flavor compounds, with 29 compounds detected and a total volatile content of 93.85 ± 7.53 μg/kg (a 32.7% increase over the Control group at day 7), both being the highest among all groups. In comparison, the Control group had only 12 volatile compounds, with a total content of 70.73 ± 8.81 μg/kg, while the DACN818 group yielded 17 compounds, with a total content of 82.60 ± 7.81 μg/kg.

**Table 1:** Volatile flavor compounds on day 7.

Variety	Compounds	CAS	RT	RI	Content of Control Group (μg/kg)	Content of DACN818 Group (μg/kg)	Content of Se-DACN818 Group (μg/kg)
Acetal	Ethane, 1,1-diethoxy-	105–57-7	3.56	726	0.31 ± 0.07	–	–
Alcohol	1-Propanol	71–23-8	1.73	556	–	3.54 ± 0.25	1.77 ± 0.27
	1-Hexanol	111–27-3	7.22	868	0.28 ± 0.02	–	–
	1-Hexanol, 4-methyl-	818–49-5	10.45	953	–	–	0.78 ± 0.34
	1-Octanol	111–87-5	13.65	1070	–	–	1.21 ± 0.17
	Phenylethyl Alcohol	1960-12-8	14.92	1116	–	4.60 ± 0.85	6.19 ± 1.17
Aldehyde	Pentanal	110–62-3	2.93	700	–	1.04 ± 0.19	–
	Hexanal	66–25-1	5.14	801	0.75 ± 0.03	0.85 ± 0.48	0.95 ± 0.73
	2-Heptenal, (*E*)-	18,829–55-5	9.95	958	–	3.60 ± 1.62	4.18 ± 1.21
	2,4-Heptadienal, (E,E)-	4313-3-5	11.77	1012	–	–	5.78 ± 3.20
	Benzeneacetaldehyde	122–78-1	12.75	1045	–	–	0.82 ± 0.05
	2-Octenal, (E)-	2548-87-0	13.22	1060	–	–	2.41 ± 0.34
	Nonanal	124–19-6	14.64	1104	–	3.27 ± 0.95	4.56 ± 1.34
	2,6-Nonadienal, (E,*E*)-	17,587–33-6	16.23	1153	–	–	0.15 ± 0.03
	Benzaldehyde, 3-ethyl-	34,246–54-3	16.54	1168	0.36 ± 0.07	–	–
	Benzaldehyde, 3,4-dimethyl-	5973-71-7	18.03	1236	–	26.49 ± 4.37	19.72 ± 4.35
	Decanal	112–31–2	18.1	1206	–	–	0.94 ± 0.14
Alkane	Hexadecane	544–76-3	25.17	1600	–	–	0.37 ± 0.15
Carboxylic acid	Acetic acid	64–19-7	1.95	610	14.22 ± 5.36	–	–
Ester	Ethyl Acetate	141–78-6	2.06	612	–	2.36 ± 1.24	–
	n-Propyl acetate	109–60-4	3.24	708	–	0.33 ± 0.04	–
	Propanoic acid, 2-hydroxy-, ethyl ester	97–64-3	5.57	815	–	–	2.55 ± 0.14
	Propanoic acid, 2-hydroxy-, propyl ester	616–09-1	8.51	918	–	0.75 ± 0.30	–
	2-Hexenoic acid, methyl ester	2396-77-2	10.25	965	28.65 ± 6.09	–	–
	Octanoic acid, methyl ester	111–11–5	13.09	1126	0.63 ± 0.31	–	0.39 ± 0.02
	Methyl salicylate	119–36-8	17.73	1192	0.54 ± 0.24	–	–
	Benzenepropanoic acid, ethyl ester	2021–28-5	21.5	1353	–	0.22 ± 0.03	0.29 ± 0.05
	2(3H)-Furanone, dihydro-5-pentyl-	104–61-0	21.74	1364	2.66 ± 1.01	–	–
	Phosphonofluoridic acid, methyl-, nonyl ester	211,192–74-4	22.24	1470	–	6.51 ± 4.66	10.68 ± 5.34
Heterocycle	Furan, 2,3-dihydro-4-methyl-	34,314–83-5	4.09	761	–	0.22 ± 0.06	0.31 ± 0.07
	1H-Indole, 1-methoxy-	54,698–11–2	19.9	1357	–	–	0.27 ± 0.03
Isothiocyanate	Allyl Isothiocyanate	1957-6-7	7.61	885	1.31 ± 0.12	8.38 ± 2.18	–
	Benzene, (2-isothiocyanatoethyl)-	2257-9-2	23.49	1469		–	3.74 ± 0.54
Ketone	1-Penten-3-one	1629-58-9	2.79	682	–	–	0.24 ± 0.12
	1-Propanone, 1-cyclopropyl-	6704-19-4	4.4	831	–	0.98 ± 0.18	0.66 ± 0.22
	1-Hepten-3-one	2918-13-0	10.73	881	–	–	0.73 ± 0.44
	5-Hepten-2-one, 6-methyl-	110–93-0	10.97	986	–	1.44 ± 0.44	3.37 ± 0.49
	2-Undecanone	112–12-9	19.48	1294	18.16 ± 3.10	–	–
	5,9-Undecadien-2-one, 6,10-dimethyl-, (E)-	3796-70-1	23.23	1453	–	–	0.19 ± 0.07
Nitrile	3-Butenenitrile	109–75-1	2.47	658	–	–	1.36 ± 0.31
	Benzenepropanenitrile	645–59-0	19.1	1245	2.86 ± 0.97	18.02 ± 3.01	18.31 ± 3.30
Phenol	Phenol, 4-ethyl-2-methoxy-	2785-89-9	20.06	1282	–	–	0.87 ± 0.10
Total					70.73 ± 8.81	82.60 ± 7.8	93.85 ± 7.53

**Fig. 5:**
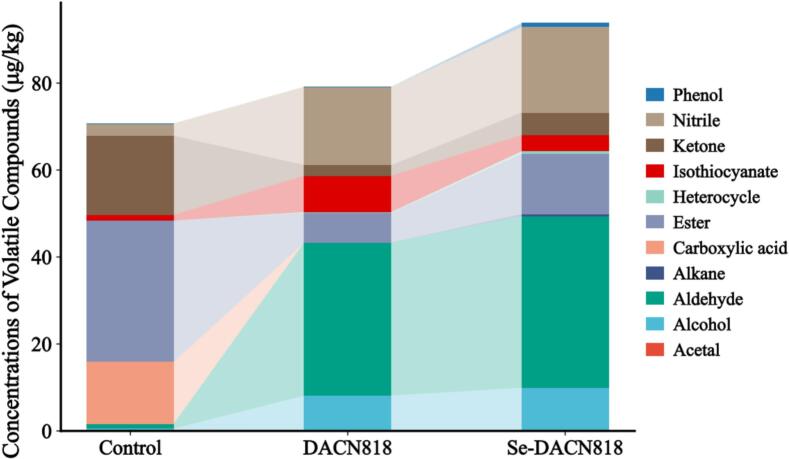
Volatile flavor compound concentration.

Further analysis of flavor profiles (Fig. 5) revealed that the Control group was dominated by esters, ketones, and carboxylic acids, with key flavor substances including acetic acid (sour),methyl 2-hexenoate (fruity), and 2-undecanone (fruity). (Api et al., 2019; Barbey et al., 2021; Bouchez & De Vuyst, 2022) In the DACN818 group, the concentrations of esters, ketones, and carboxylic acids declined, while aromatic aldehydes (including 3,4-dimethylbenzaldehyde) and nitriles (primarily benzenepropanenitrile) increased significantly. Given that aromatic aldehydes are associated nutty, almond-like notes, and benzenepropanenitrile contributes aldehydic and spicy notes, their enrichment likely improved the sensory profile of this group. (Li, Guan, et al., 2023; Wang et al., 2025) The Se-DACN818 group not only maintained the aldehyde and nitrile levels observed in the DACN818 group but also showed increased contents of fruit-associated flavor compounds such as alcohols (phenylethyl alcohol), ketones (6-methyl-5-hepten-2-one), and esters (ethyl 2-hydroxypropanoate). (Antonova-Nedeltcheva et al., 2025; Yu et al., 2025; Zhou, Wang, et al., 2021).

Isothiocyanates (ITCs, typical pungent flavor compounds in cruciferous vegetables) mainly include allyl isothiocyanate (AITC) and phenethyl isothiocyanate (PEITC). When the cellular structure of cruciferous vegetables is disrupted, endogenous myrosinase comes into contact with glucosinolates and hydrolyzes them, leading to ITC formation. (Narra et al., 2025) In this study, the ITC contents in the DACN818 and Se-DACN818 groups was approximately 6.5-fold and 3-fold higher than that in the Control group, respectively, indicating a marked increase. A trend consistent with observations in Zhacai fermented with *Lactiplantibacillus plantarum* LPP95. (Tan et al., 2024) Importantly, the Se-DACN818 group exhibited substantially lower ITC levels (a 55.37% reduction) compared to the DACN818 group, suggesting that Se-DACN818 can suppress ITC formation during LAB fermentation, thereby mitigating the pungency of fermented Zhacai.

### 3.8 Untargeted metabolomics analysis

**Fig. 6:**
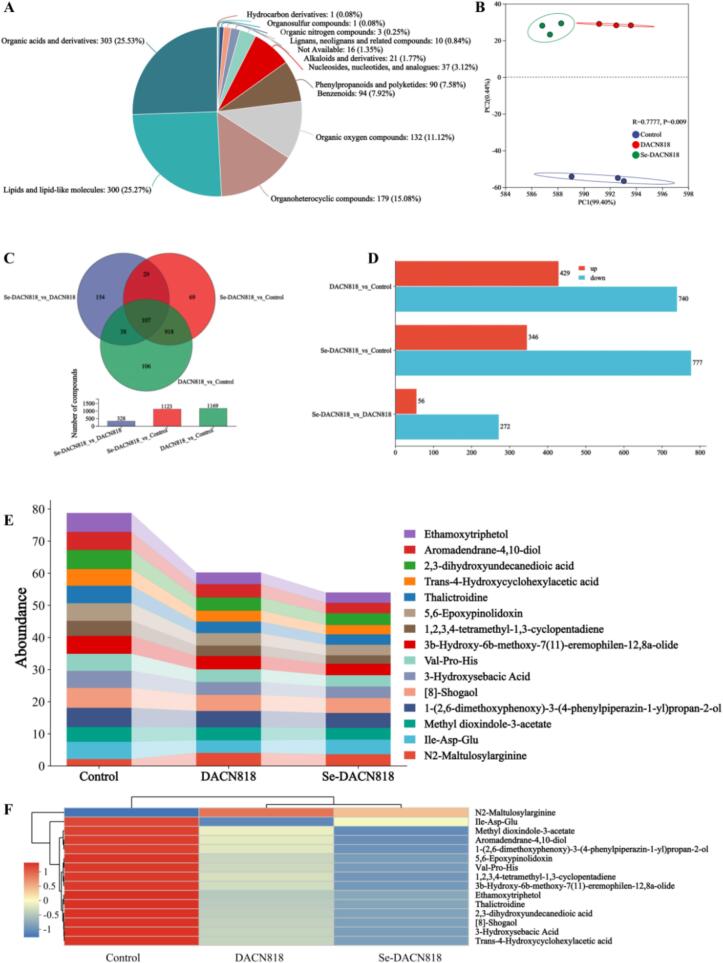
Untargeted metabolomics analysis of Zhacai on day 7. (A) Metabolite categories in mix mode; (B) PCA; (C) Venn diagram; (D) Statistics of upregulated and downregulated metabolites; (E) DAM abundance; (F) Hierarchical clustering heatmap of DAMs.

Classification against the HMDB database identified a total of 1187 metabolites. The most abundant class was that of lipids and lipid-like molecules (300 metabolites, 25.27%), followed by organoheterocyclic compounds (179 metabolites, 15.08%), organic acids and derivatives (132 metabolites, 11.12%), and organic oxygen compounds (132 metabolites, 11.12%) (Fig. 6A). Principal component analysis (PCA) revealed a clear separation in metabolite profiles between the DACN818 and Se-DACN818 groups (Fig. 6B). (Fan et al., 2023; Li et al., 2024) Pairwise comparisons identified a total of 1421 differentially abundant metabolites (DAMs) (Fig. 6C). Specifically, compared to the Control group, 1169 DAMs were detected in the DACN818 group (429 upregulated, 740 downregulated), while 1123 DAMs were found in the Se-DACN818 group (346 upregulated, 777 downregulated) (Fig. 6D). Notably, a direct comparison between the Se-DACN818 and DACN818 groups showed that 272 of the 328 DAMs were downregulated.

Further analysis of the 15 key DAMs (VIP > 1.5) through screening (Fig. 6E, F) indicated that their overall abundance was higher in the Control group compared to the DACN818 and Se-DACN818 groups, and most of these metabolites showed an even more pronounced reduction in the Se-DACN818 group. Notably, the abundance of N2-maltulosylarginine, which is associated with aroma formation, increased significantly in both the DACN818 and Se-DACN818 groups. (Shakoor et al., 2022) The abundance of Ile-Asp-Glu, a tripeptide associated with umami taste, was also elevated in the Se-DACN818 group. (Dutta et al., 2022; Yang et al., 2024)

### 3.9 Microbial community analysis

**Fig. 7:**
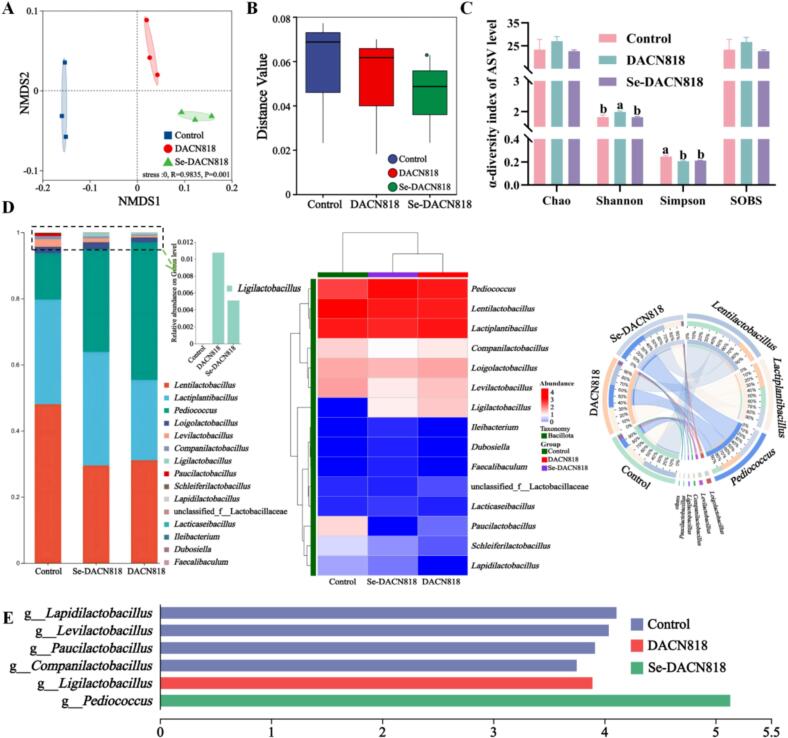
Microbial community analysis of Zhacai on day 7. (A) NMDS at the ASV level; (B) Analysis of β-diversity differences between groups; (C) α-diversity indices; (D) Microbial community composition; (E) LEfSe analysis.

Non-metric multidimensional scaling (NMDS) analysis (Fig. 7A) revealed that inoculation with DACN818 and Se-DACN818 significantly altered the microbial community structure of Zhacai, which is consistent with previous findings in inoculated fermented vegetables. (Xu et al., 2024) Analysis of β-diversity differences between.

groups (Fig. 7B) further showed that the Bray-Curtis distances among samples within the inoculated groups were smaller than those in the Control group, indicating a more stable microbial composition. This may be attributed to the suppression of random growth of some indigenous microorganisms by the inoculants. (Chen et al., 2020) Notably, the distance values in the Se-DACN818 group were lower than those in the DACN818 group, suggesting that selenium enrichment further enhanced the stability of the community structure, which may contribute to more consistent fermentation performance. As shown in Fig. 7C, the Shannon index of the DACN818 group was significantly higher than that of the Control and Se-DACN818 groups, while the Simpson indices of both inoculated groups were lower than that of the Control, indicating that DACN818 and Se-DACN818 differentially affected species diversity and evenness, overall reducing community evenness.

As shown in Fig. 7D, inoculation with either DACN818 or Se-DACN818 altered the microbial community composition, and a clear difference was also evident between the two inoculated groups. *Lentilactobacillus* was the dominant genus in the Control group (47.94%), but its relative abundance decreased to approximately 30% in DACN818 and Se-DACN818 groups. In contrast, the abundance of *Pediococcus* increased from 14.04% in the Control group to 31.16% and 41.59% in the DACN818 and Se-DACN818 groups, respectively, making it as the dominant genus. Notably, despite the inoculation with the *Ligilactobacillus* strain, the relative abundance of this genus was low in the DACN818 and Se-DACN818 groups at the later fermentation stage. The selective promotion of certain *Lactobacillus* and inhibition of other naturally dominant strains by inoculation with specific LAB has been previously demonstrated. (Cao et al., 2022; Xu et al., 2024) These results suggested that the regulation of the fermentation community was not a simple result of colonization by the inoculated strain, but rather involved complex interspecific interactions. Specifically, DACN818 inoculation significantly enriched *Pediococcus*, and Se-DACN818 further enhanced this effect.

Linear discriminant analysis effect size (LEfSe) analysis (Fig. 7E) indicated that *Levilactobacillus*, *Paucilactobacillus*, *Lapidilactobacillus*, and *Companilactobacillus* had high LDA scores in the Control group, suggesting these as characteristic taxa. In the DACN818 group, *Ligilactobacillus* showed a high LDA score, while *Pediococcus* had the highest LDA score in the Se-DACN818 group. These differentially abundant taxa are considered key drivers of the inter-group microbial community differences, which was consistent with the observed changes in the abundance of the dominant genera.

### 3.10 Correlation analysis between microbial communities and quality indicators

**Fig. 8:**
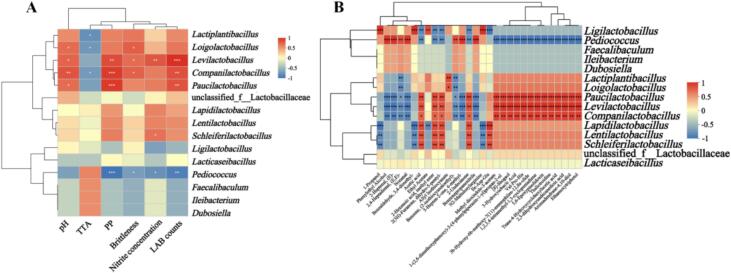
Correlation analysis. (A) Correlations between microbial genera and physicochemical indicators; (B) Correlations between microbial genera and volatile/non-volatile metabolites.

As shown in Fig. 8A, nitrite content correlated negatively with *Pediococcus* but positively with *Levilactobacillus* and *Companilactobacillus*. Texture brittleness and PP content were negatively correlated with *Pediococcus,* but positively correlated with *Levilactobacillus*, *Companilactobacillus*, and *Paucilactobacillus*. Previous studies have often observed consistent trends between LAB, acidification, nitrite reduction, and decreased texture brittleness. (Huang et al., 2021; Li et al., 2025) However, in this study, some LAB genera showed a positive correlation with nitrite, while others were correlated with pectin content and texture brittleness.

This may be due to differences in the microbial composition and the substrate. (Lin & Peddada, 2023) Nevertheless, it is noteworthy that the correlation trends of *Ligilactobacillus* with multiple indicators (such as PP, nitrite content, viable bacterial count) were similar to those of *Pediococcus*, which may be related to the inoculation of *Ligilactobacillus* strains DACN818 and Se-DACN818 promoting the proliferation of *Pediococcus*.

Correlation analysis between metabolites and microbial genera (Fig. 8B) revealed that Ile-Asp-Glu was significantly negatively correlated with *Ligilactobacillus*, which may explain its elevated level in the Se-DACN818 group. Several key flavor components in Zhacai, including 1-propanol, 3,4-dimethylbenzaldehyde, ethyl acetate, and N2-maltulosylarginine, were significantly positively correlations with *Ligilactobacillus*, suggesting that these may be characteristic metabolites of this genus. Additionally, numerous volatile metabolites (such as phenylethyl alcohol and (E)-2-heptenal) showed positive correlation with *Pediococcus*, whereas several non-volatile metabolites (such as methyl dioxindole-3-acetate, [8]-shogaol, thalictrine and aromaden-diacetic acid) were negatively correlated. These findings implied that *Pediococcus* is closely associated with the increase in volatile compounds and the concurrent decrease in non-volatile metabolites observed in the Se-DACN818 group. Moreover, *Paucilactobacillus*, *Levilactobacillus*, and *Companilactobacillus* displayed similar correlation patterns with metabolites, indicating that they may share similar metabolic pathways.

## 4 Conclusion

Selenium enrichment of *Ligilactobacillus salivarius* DACN818 partially enhanced both the safety and flavor profile of Zhacai. Specifically, the application of Se-DACN818 effectively reduced nitrite content by modulating the microbial community structure, increasing the relative abundance of *Pediococcus* while decreasing that of *Levilactobacillus*. Moreover, it increased the variety and total content of volatile flavor compounds, promoting the accumulation of key flavor substances such as 1-propanol, ethyl acetate, and N2-maltulosylarginine. These changes had a significant positive correlation with the presence of *Ligilactobacillus*. Nevertheless, while Se-DACN818 suppressed the formation of pungent isothiocyanates compared to DACN818, it also reduced brittleness and PP content, accompanied by microstructural alterations in Zhacai. However, the specific mechanisms driving these changes remain unclear. Therefore, future studies should focus on elucidating the deterioration mechanism of texture in Zhacai caused by Se-DACN818 while simultaneously enhancing its safety and flavor. These will support its rational application in fermented foods.

## CRediT authorship contribution statement

**Qingxu Guo:** Writing – original draft, Methodology, Investigation, Formal analysis, Conceptualization. **Jiajing Wang:** Writing – original draft, Project administration, Formal analysis. **Yue Li:** Investigation, Data curation. **Yi Wu:** Visualization, Investigation. **Dejun Liu:** Resources, Methodology. **Xiaoyong Chen:** Writing – review & editing, Supervision, Project administration, Funding acquisition.

## Declaration of competing interest

The authors declare that they have no known competing financial interests or personal relationships that could have appeared to influence the work reported in this paper.

## Data Availability

Data will be made available on request.
